# The emerging antioxidant paradigm of mesenchymal stem cell therapy

**DOI:** 10.1002/sctm.19-0446

**Published:** 2020-06-04

**Authors:** Rhian Stavely, Kulmira Nurgali

**Affiliations:** ^1^ Institute for Health and Sport, Victoria University, Western Centre for Health, Research and Education, Sunshine Hospital Melbourne Victoria Australia; ^2^ Department of Pediatric Surgery Massachusetts General Hospital, Harvard Medical School Boston Massachusetts USA; ^3^ Department of Medicine Western Health, Faculty of Medicine, Dentistry and Health Sciences The University of Melbourne Melbourne Victoria Australia; ^4^ Regenerative Medicine and Stem Cells Program Australian Institute of Musculoskeletal Science (AIMSS) Melbourne Victoria Australia

**Keywords:** antioxidant, mesenchymal stem cell, mitochondria, multipotent stromal cell, oxidative stress, reactive oxygen species

## Abstract

Mesenchymal stem cells (multipotent stromal cells; MSCs) have been under investigation for the treatment of diverse diseases, with many promising outcomes achieved in animal models and clinical trials. The biological activity of MSC therapies has not been fully resolved which is critical to rationalizing their use and developing strategies to enhance treatment efficacy. Different paradigms have been constructed to explain their mechanism of action, including tissue regeneration, trophic/anti‐inflammatory secretion, and immunomodulation. MSCs rarely engraft and differentiate into other cell types after in vivo administration. Furthermore, it is equivocal whether MSCs function via the secretion of many peptide/protein ligands as their therapeutic properties are observed across xenogeneic barriers, which is suggestive of mechanisms involving mediators conserved between species. Oxidative stress is concomitant with cellular injury, inflammation, and dysregulated metabolism which are involved in many pathologies. Growing evidence supports that MSCs exert antioxidant properties in a variety of animal models of disease, which may explain their cytoprotective and anti‐inflammatory properties. In this review, evidence of the antioxidant effects of MSCs in in vivo and in vitro models is explored and potential mechanisms of these effects are discussed. These include direct scavenging of free radicals, promoting endogenous antioxidant defenses, immunomodulation via reactive oxygen species suppression, altering mitochondrial bioenergetics, and donating functional mitochondria to damaged cells. Modulation of the redox environment and oxidative stress by MSCs can mediate their anti‐inflammatory and cytoprotective properties and may offer an explanation to the diversity in disease models treatable by MSCs and how these mechanisms may be conserved between species.

## Graphical abstract

MSCs exhibit antioxidant properties directly or indirectly. These antioxidant effects of MSCs associate with their therapeutic benefit in an array of pathologies.

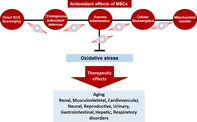

**Significance statement**

The role of mesenchymal stem cells (MSCs) in ameliorating oxidative and nitrosative injury has received considerable attention in recent years. The reduction‐oxidation (redox) environment regulates many physiological and pathophysiological mechanisms in cellular biology. Oxidative stress and redox imbalance are mediated by molecular constituents that are present in all living cells and share similar functions. The ability of MSCs to regulate these processes may offer an explanation to the diversity of disease models treatable by MSCs and to the effects of MSCs conserved between species. In this review, evidence of direct and indirect antioxidant mechanisms of MSC therapies is explored.

## 1 INTRODUCTION

Mesenchymal stem cells (multipotent stromal cells; MSCs) have been used as tools to treat a broad range of diseases in animal models due to their unique characteristics such as host immune evasion, rapid expansion, and their affluence in adult bone marrow and adipose tissue. The positive outcomes of these studies have driven hundreds of clinical trials into their application for diabetes, inflammatory disorders and various liver, kidney, lung, cardiovascular, musculoskeletal, neurological, and gastrointestinal diseases.^1^ While several trials have demonstrated the therapeutic potential of MSCs, the failure to incorporate MSCs into current treatment regimens can be, in part, attributed to the lack of understanding pertaining to their biological mechanisms of action.

Initially, MSCs were explored as tools of regenerative medicine to replace damaged tissue.^2^ However, administered MSCs were rarely observed to differentiate and effectively engraft into host tissues despite demonstrating favorable effects in many disease models.^3^ Furthermore, the secretome of MSCs was identified to be therapeutic in many disease models in vitro and in vivo. Together, this resulted in a paradigm shift in recognition of the trophic actions of MSCs.^4^ Despite extensive research investigating the anti‐inflammatory and trophic constituents of the MSC‐derived secretome, the therapeutic mechanisms of MSCs remain incompletely resolved.^5^ MSCs demonstrate therapeutic attributes across xenogeneic barriers and, therefore, the therapeutic mechanisms of MSCs may be similar between species. There is strong evidence that the effects of MSCs are mediated via the secretion of protein/peptide ligands; however, it is equivocal whether these ligands are effective across xenogeneic barriers.

**FIGURE 1:**
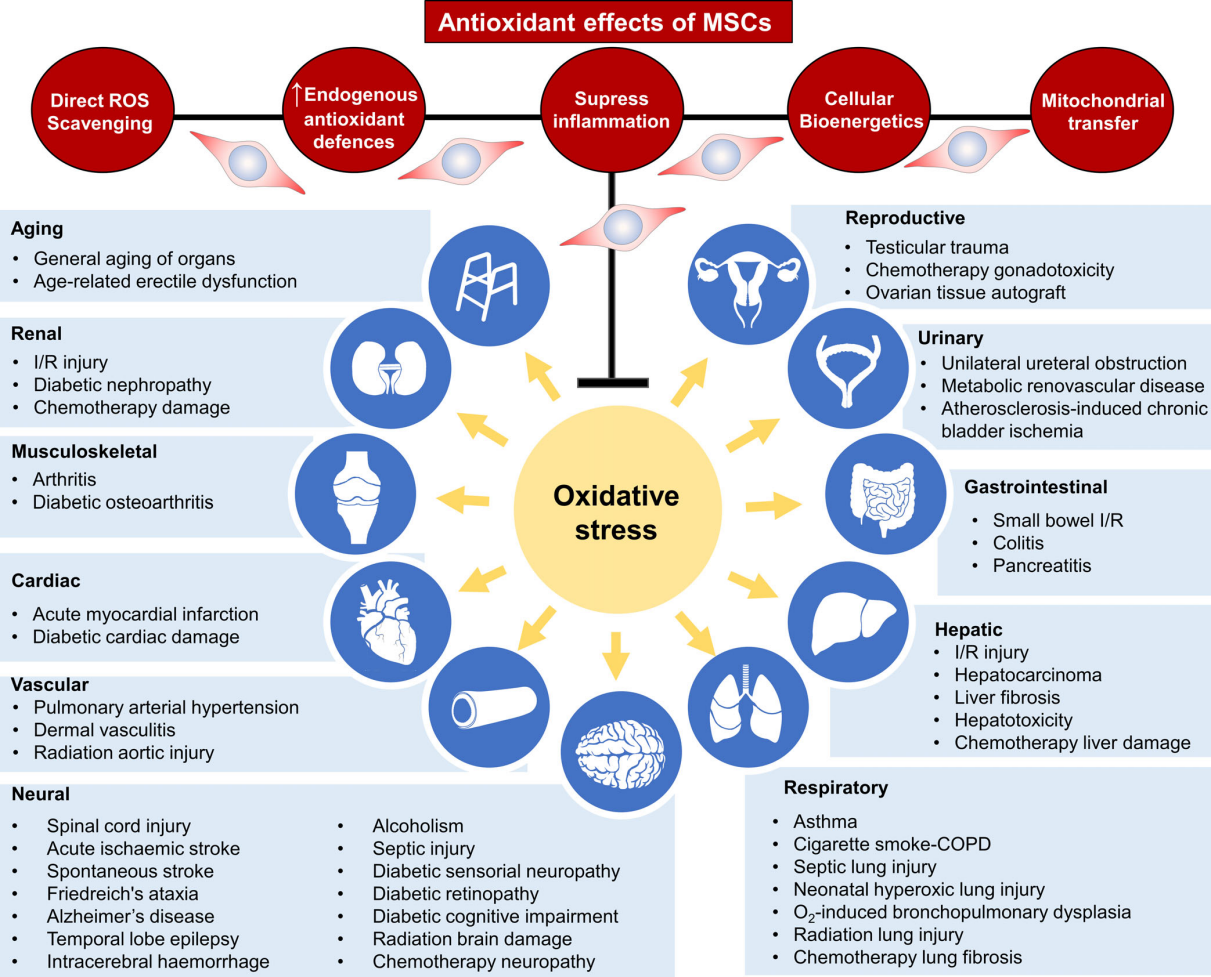
Antioxidant effects of MSC therapies. MSCs exhibit antioxidant properties directly by scavenging of free radicals and donating mitochondria or indirectly by upregulation antioxidant defenses in other cells and altering cellular bioenergetics. The immunosuppressive properties of MSCs can also avert the generation of reactive oxygen species (ROS). These mechanisms reduce oxidative stress, which associates with the therapeutic benefit of MSCs in an array of pathologies

Recently, the role of MSCs in ameliorating oxidative and nitrosative injury has received considerable attention. The reduction‐oxidation (redox) environment regulates many physiological and pathophysiological mechanisms in cellular biology. Antioxidant effects of MSC therapy have been observed in various disease models such as diabetic injuries to the kidney, retina, sensory neurons, brain, and bone formation; chemotherapy‐ or radiation‐induced injury to the lungs, gonads, aorta, and brain; ischemic injury of the brain, heart, kidney, and liver; and traumatic injury to the spine and testis, cognitive disorders, gastrointestinal inflammation, septic injuries, and aging (Figure 1; Table 1). MSCs can directly reduce oxidative stress‐related injury in vitro in glial cells, neurons, cardiomyocytes, renal cells, endothelial cells, immune cells, hepatocytes, islet cells, fibroblasts, skeletal muscle, and other cells (Table 2). Oxidative stress is concomitant with cellular injury, inflammation, and dysregulated metabolism and, therefore, is a key pathophysiological mechanism of many diseases. Oxidative stress and redox imbalance are mediated by molecular constituents that are present in all living cells and share similar functions. Thus, the ability of MSCs to regulate these processes may offer an explanation to the diversity of disease models treatable by MSCs and to the effects of MSCs conserved between species.

**TABLE 1:** Antioxidant activity of MSCs in disease models

Application	Model	MSCs used	Effects of MSC treatment	Antioxidant mechanisms	References
Aging	Aging‐related erectile dysfunction (rat)	Rat AT‐MSCs	↑ Erectile response	↓ Lipid peroxidation ↑ SOD activity	62
	Premature aging (Bmi−/−) (mouse)	Mouse amniotic membrane MSCs	↑ Survival time ↓ Apoptosis in thymus and kidney ↑ Proliferation in thymus and kidney ↑ Mature immune cells ↑ Skeletal muscle growth ↓ Osteoporosis ↑ Bmi‐1 in liver, kidney, thymus, muscle, spleen, lung, and bone marrow	↓ H_2_O_2_, ↑CAT, ↑SOD in the heart, liver, spleen, lung, kidney, BM and thymus, ↓ ROS in all except heart. ↓ DNA damage in cells of BM, spleen, lung and thymus MSCs secrete SOD (total) and CAT	33
Chemotherapy and radiation	Bleomycin‐induced pulmonary fibrosis (rat)	Rat mesenchymal stem cells (H4320‐1)	↓ Fibrosis	↓ NRF2, ↓ NQO1, ↓ HO‐1, ↓ γ‐GCS ↓ Lipid peroxidation, ↑ SOD activity All attenuated to near control levels	63
	Bleomycin‐induced pulmonary fibrosis (mouse)	Human BM‐MSCs cell line Ue6E7T‐2	↓ Collagen	↓ DNA oxidation ↓ ER stress marker BiP Effects negated by silencing STC‐1 and enhanced by STC‐1 over expression	127
	Cisplatin‐induced acute kidney injury (rat)	Human UC‐MSC exosomes	↓ Blood urea nitrogen (MSC‐CM and fibroblast exosomes had no effect) ↓ Creatinine (MSC‐CM and fibroblast exosomes had no effect) ↓ Gross morphological damage ↓ Apoptosis (TUNEL) ↑ PCNA ↓ Bax, ↑ Bcl‐2 ↓ p38MAPK	↓ DNA oxidation ↑ GSH ↓ Lipid peroxidation	27
	Cisplatin‐induced cognitive impairment (mice)	Mouse BM‐MSCs Intranasal delivery	↑ Cognitive function	↑ Maximal respiratory capacity and spare respiratory capacity of mitochondria ↓ Morphologically atypical mitochondria	112
	Cisplatin‐induced gonadotoxicity (rat)	Rat BM‐MSCs	↑ Testis weight and testosterone levels ↓ TNFα	↓ Lipid peroxidation, ↑ SOD activity, ↑ GSH ↓ iNOS	64
	Cisplatin‐induced renal injury (mouse)	Mouse BM‐MSC‐CM	↓ Weight loss ↓ Serum creatinine levels ↓ c‐caspase 3 expression ↓ Gross morphological damage	HO‐1 ^−/−^ MSCs did not demonstrate therapeutic value	94
	Lung radiation injury (mouse)	Mouse aorta‐derived and BM‐MSCs	↓ Lung fibrosis	Aorta and BM‐MSCs secrete SOD1 ↑ SOD1 expression in irradiated lung SOD1 mimetic replicated effect of MSCs	59
	Paclitaxel‐induced neuropathy (rat)	Rat BM‐MSCs	↑ Responses to thermal hyperalgesia and cold allodynia Sciatic nerve: ↑ NGF ↓ Pro‐inflammatory cytokines ↓ c‐caspase 3	Sciatic nerve: ↑ Total antioxidant capacity	55
	Radiation‐induced aortic injury (mouse)	Human BM‐MSCs	↓ Aorta thickness ↓ Collagen ↓ TGFβ ↓ TNFα ↓ ICAM ↓ Apoptosis	↓ Nitrotyrosine ↓ Lipid peroxidation, ↑ HO‐1 ↑ CAT	82
	Radiation‐induced neurological complication (mouse)	Human AT‐MSCs	↑ Cognitive function ↓ Neuron loss ↓ Caspase 3	↓ Lipid peroxidation in hippocampus and brain lateral ventricle ↓ iNOS	41
Hyperglycemic injuries	Alloxan‐induced diabetes (rat)	Rat BM‐MSCs	↑ Insulin ↓ Glucose levels ↓ Total cholesterol ↓ Triglycerides ↑ Vitamin E	↑ GSH, ↑ GST, ↑ SOD ↓ NO ↓ lipid peroxidation	88
	Db/Db mouse model of type 2 diabetes	Mouse amniotic fluid MSCs	Improved kidney function ↓ Weight gain ↓ Pro‐inflammatory cytokines↓ Apoptosis Overexpression of Sirtuin3 in MSCs improved all effects	↓ Lipid peroxidation ↓ 8‐isoprostane ↑ GSH ↓ GSSG	85
	Diabetes‐induced cognitive impairment (mouse)	Rat BM‐MSC and exosomes	↑ Cognitive function Hippocampus (CA1): No change in neuronal numbers Exosomes colocalized with astrocytes and can be detected in microglia and neurons	↓ Lipid peroxidation	26
	Diabetic retinopathy (mouse)	Mouse AT‐MSCs Intravitreal injection	↓ Retinal ganglion cell loss ↑ NGF, bFGF and GDNF ↑ TSP1	↓ ROS and lipid peroxidation	28
	STZ‐induced diabetic osteoarthritis (mouse)	Mouse AT‐MSCs	↑ Chondrocytes ↓ RAGE, NFκB	↓ Lipid peroxidation	128
	STZ‐induced diabetic nephropathy (rat)	Rat BM‐MSCs	↓ Urinary albumin excretion and ameliorated glomerulosclerosis	↓ Lipid peroxidation ↓ ROS ↑ SOD activity ↓ GLUT1	29
	STZ‐induced sensorial diabetic Neuropathy (mouse)	Mouse BM‐MSCs	Improved pain‐like behaviors ↓ IL‐1β ↓TNFα ↑IL‐10 ↑ TGFβ ↓ Galectin‐3	↓ Lipid peroxidation ↓ Nitrite ↓ CAT ↓Gpx ↓ Nrf2 mRNA	40
	STZ‐nicotinamide (diabetes)‐induced cardiac damage (rat)	Rat BM‐MSCs	Normalization of gene expression associated with cardiac glucose and fatty acid uptake (IRS‐1, GLUT4, PPARα, PGC‐1, CPT1a and SREBP‐1c) ↓ c‐caspase 3, Bax and ↑ Bcl‐2 ↓ Cardiac fibrosis	↓ Total oxidant content in serum ↑ Total antioxidant capacity in heart ↓ MPO ↓ iNOS	56
Inflammation	Caecal ligation‐puncture induced sepsis (rat)	Rat AT‐MSCs Administration of serum‐starved MSCs	↑ Apoptosis in serum‐starved MSC↓ TNFα, NFkB in lungs and kidney ↓ Mitochondrial Bax and ↑ Bcl‐2 in lungs and kidney “Healthy” MSCs had no effect	↓ Protein oxidation in kidney ↑ NQO1 and HO‐1 in the lungs	92
	DSS‐induced colitis (mouse)	Mouse BM‐MSCs	↓ Mucosal permeability: D‐lactic acid and Diamine oxidase ↑ E‐cadherin	**↓** Lipid peroxidation ↑ SOD	65
	*E coli*‐induced Acute lung injury (mouse)	Mouse BM‐MSCs	↓ Edema	↓ MPO activity ↑ CAT, SOD, GPx, glutathione reductase and GSH ↑ Total antioxidant capacity ↓ Lipid peroxidation ↓ iNOS expression	37
			More favorable results (↓morphological lung damage, iNOS expression and lipid peroxidation) with MSCs administered 24 h Pre‐*E coli* exposure than 12 hours post‐treatment		
	Endotoxin‐induced inflammation in plasma (mouse)	Mouse BM‐MSCs	↑ Cys but ND to lung fibroblast Redox homeostasis (6 hours) superseded ↓IL‐1β and ↓TNFα (2 hours)	↑ GSH ND to glutathione disulfide (GSSG) or cysteine (CySS) ↓ GSH/GSSG redox potential ↓ Cys/CySS redox potential, ND to lung fibroblast	51
	Freund's adjuvant‐induced arthritis (rat)	Rat BM‐MSCs	↓ Antinuclear antibodies ↓ TNFα, IL‐9 and IL‐4 ↑ IFNγ and TGFβ ↓ Immune cell infiltration ↓ Cartilage and bone loss	↓ Lipid peroxidation ↑ GSH↑ SOD activity	66
	HOCl‐induced systemic sclerosis (mouse)	Mouse BM‐MSCs	Serum: ↓ Systemic sclerosis biomarker (SCL‐70) Skin and lung: ↓ Collagen, ↓ αSMA ↓ TGFβ1	↓ Advanced oxidation protein products ↑ Total antioxidant capacity	57
	IL‐10 −/− model of colitis (mouse)	Human BM‐MSCs	↓ TNFα, IFNγ, IL‐4 and p‐NFκB	↓ O_2_^.−^ and H_2_O_2_ ↓ Lipid peroxidation ↑ SOD1 and CAT	34
	Immune complex‐mediated dermal vasculitis (mouse)	Human AT‐MSCs	↓ Neutrophil accumulation ↓ Vascular permeability	Effects dependent on SOD3 expression by MSCs	79
	LPS‐induced lung injury (rat)	Rat BM‐MSCs	↓ Lung edema ↓ Bronchoalveolar lavage protein ↓ Bronchoalveolar lavage cells ↓ Neutrophils ↓ NFκB, ↑ IL‐10	↓ MPO ↓ Lipid peroxidation ↓ FASL	107
	Sepsis‐induced brain injury (rat)	Rat AT‐MSC exosomes	↓ Apoptosis ↓ Inflammatory markers	↓ Protein oxidation ↓ NOX1‐2	25
	Severe acute pancreatitis (rat)	Human BM‐MSCs	↓ Serum amylase and lipase ↓ Pancreatic damage ↓ Inflammatory cells MSCs migrated to tissue stimulated: ↓ Pro‐inflammatory cytokines: TNFα, IL‐1β, IL‐6	MSCs migrated to tissue stimulated: ↓ Lipid peroxidation ↑ SOD activity, ↑ GPx ↓ iNOS	67
	Severe acute pancreatitis (rat)	Rat BM‐MSCs	↓ Pancreatitis score Inhibition of HO‐1 by zinc protoporphyrin partially negated the effects of MSCs	↑ HO‐1 ↑ CO ↓ MPO ↓ ROS and lipid peroxidation ↑ SOD activity and CAT expression	30
Kidney and bladder disease	Atherosclerosis‐induced chronic bladder ischemia (rat)	Human amniotic fluid‐derived MSCs	↓ Bladder overactivity ↓ TNFα	↓ DNA oxidation ↓ Lipid peroxidation	129
	Metabolic renovascular disease in swine	Swine AT‐MSC extracellular vesicles	↑ Capillary density	Extracellular vesicles contained antioxidant proteins: Glutathione Peroxidase 1,4,6,7, GST Zeta 1, SOD1‐3, Peroxiredoxin 1‐6, Catalase, Cold Shock Domain Containing E1, Cytoglobin, Microsomal GST 3, Prostaglandin‐Endoperoxide Synthase 1, Peroxidasin, Albumin, Apolipoprotein E, Glutathione‐Disulfide Reductase, Thioredoxin Reductase 1‐2 ↓ 8‐isoprostane ↓ ROS ↓ Nitrotyrosine	31
	Unilateral ureteral obstruction (rat)	Human UC‐MSC‐CM	↓ Renal tubular damage ↓ Fibrosis ↓ Apoptosis ↑ Cell proliferation	↓ ROS ↓ Lipid peroxidation ↑ GSH	24
Liver disease	Acetaminophen‐induced acute liver failure (mouse)	Human UC‐MSCs	MSC pretreatment and post‐treatment of induced liver in injury ↑ Survival and liver weight ↓ Biomarkers of liver failure ↓ Apoptotic cells and necrotic tissue ↓ IL‐6 only observed with pretreatment	↑ GSH ↑ SOD activity ↓ Lipid peroxidation only observed with pretreatment	38
	CCl^4^‐induced liver fibrosis (mouse)	Human BM‐MSCs	↓ p47‐phox cells	↓ Lipid peroxidation ↑ SOD activity, CAT and GSH	68
	CCl^4^‐induced liver injury (mouse)	Allogeneic BM‐MSC	↑ Serum albumin ↓ Serum ALT and AST ↓ Expression of TNFα, IL‐6, type 1 collagen and αSMA MSCs outperformed hematopoietic stem cells in all assays	↓ MPO ↑ SOD and catalase ↓ Lipid peroxidation	50
	CCl^4^–induced liver injury (rat)	Human BM‐MSC	↓ Serum ALT and AST ↓ Liver fibrosis	↑ GSH ↓ Lipid peroxidation	89
	CCl^4^‐induced rat liver fibrosis	Human BM‐MSCs (cells and exosomes)	↓ Fibrosis ↓ Pro‐inflammatory cytokines ↓Wnt signaling	↓ Lipid peroxidation	130
	N‐diethylnitrosamine‐induced hepatocarcinoma (rat)	Rat BM‐MSCs	Administration of MSCs at early stage: ↓ Tumor incidence ↓ Tumor volume Administration of MSCs at late stage: ↑ Tumor volume	↓ Lipid peroxidation ↓ Mitochondrial O_2_^.−^ ↑ Total antioxidative capacity ↓ DNA damage	36
	Thioacetamide‐induced liver injury (mouse)	Canine BM‐MSCs	↓ Lung injury ↓ Fibrosis	↑ Total antioxidant capacity ↓ Lipid peroxidation	58
Lung diseases	Cigarette smoke‐induced chronic obstructive pulmonary disease (guinea pig)	Guinea pig AT‐MSCs IV and intratracheal delivery	No effect on emphysema score	↑ Thiol after IV administration ↓ Lipid peroxidation after IV and intratracheal delivery	131
	Mustard lung (human case study)	Human AT‐MSCs	Functional respiratory improvement	↑ GSH in sputum ↓ Lipid peroxidation	49
	Ovalbumin and aluminum hydroxide‐induced asthma (mouse)	Human BM‐MSC	Functional recovery ↓ Mucin ↓ Collagen	↓ Nitrotyrosine	132
Neural injury and cognition	APP/PS1 transgenic model of Alzheimer's disease (mouse)	Rat AT‐MSCs	↑ Recognition in behavioral test ↑ Neurogenesis	↓ ROS	32
	Chronic ethanol intake (rats)	Human AT‐MSCs	AT‐MSCs activated by TNFα and IFNγ ↓ETOH intake ↓Relapse after ETOH deprivation	↓ Hippocampal GSSG/GSH	86
	Collagenase induced‐intracerebral hemorrhage (rat)	Rat BM‐MSCs	↓ Apoptosis ↓ Edema ↓ Blood–brain barrier permeability ↓ Pro‐inflammatory cytokines ↑ TSG6, TGFβ1 and IL‐10	↓ iNOS ↓ ONOO− ↓ MPO	39
	Pilocarpine induction of temporal lobe epilepsy (rat)	Rat BM‐MSCs	↓ Caspase 3 ↓ Glutamate ↑GABA↓TNFα ↓ IL‐1β	↑ GSH ↓ Lipid peroxidation ↑ Paraoxonase‐1	90
	Spontaneous stroke (rat)	Rat BM‐MSCs	↑ Bcl‐2 expression Prevented hippocampal lesions ↓ Apoptosis	↓ O_2_^.−^ ↓ Lipid peroxidation	35
	Tg2576 mice (Alzheimer's disease)	Human UC‐MSCs	Improved cognitive function Effects on hippocampus: No change in β‐amyloid levels ↑Neurogenesis	↓ Lipid peroxidation ↑ SOD activity ↑ nNOS ↑total NO	43
	YG8 transgenic model of Friedreich's ataxia (mouse)	Mouse BM‐MSCs	Improved performance on behavioral tests ↑ BDNF, NT3, and NT4 in dorsal root ganglia (DRG) ↑ GFAP, Tuj1, and MAP2 in DRG ↑ Bcl‐2 ↑ Frataxin	↓ SOD2 and SOD3↑ CAT and GPx1	81
Oxygen tension injuries	Acute ischemic stroke (rat)	Swine AT‐MSCs	↓ Infarct area ↓ Inflammatory cytokines ↓ c‐caspase 3 ↓ c‐PARP ↓ γ‐H2AX ↓cytosolic cytochrome *c*	↓ NOX1 and NOX2 ↓ Protein oxidation	102
	Acute myocardial infarction (swine)	Swine BM‐MSCs Autologous	↓ Bax, c‐caspase 3 and c‐PARP ↓ Inflammation ↓ Infarct area Improved echocardiography parameters	↓ Oxidized protein ↓ NOX1 and NOX2	103
	Hepatic I/R injury (rat)	Human UC‐MSC extracellular vesicles	↓ Necrotic area ↓ c‐caspase3	↓ ROS Silencing of SOD2 in MSCs inhibits therapeutic effect of extracellular vesicles SOD2 mimetic restored effects of MSC	80
	I/R model of laparoscopic partial hepatectomy (swine)	Swine AT‐MSCs	Improved hepatic biochemical markers	↓ Lipid peroxidation ↓ MPO ↑ SOD activity	69
	Kidney from acute I/R injury (rat)	Rat AT‐MSCs (cells and exosomes)	MSCs and exosomes improved kidney function ↓ Histological injury score ↓ Pro‐inflammatory cytokines ↓ TGFβ↓ c‐caspase 3, c‐PARP, mitochondrial Bax, cytochrome *c* translocation Additive effects with MSCs and exosomes	↓ NOX1 and NOX2 ↓ Oxidized protein ↓ MPO ↓ γ‐H2AX DNA damage	104
	Kidney ischemia (rat)	Human WJ‐MSC Extracellular vesicles	↓ Renal injury score ↓ Apoptosis ↑ Nuclear NRF2 Fibroblast exosomes had no effect	↓ Lipid peroxidation ↓ DNA oxidation ↑ SOD activity ↑ HO‐1 expression	52
	Myocardial I/R injury (mouse)	HuES9.E1 derived MSC exosomes	↓ Infarct size in vivo and ex vivo Ex vivo results suggest direct effect on myocardium Disrupted exosomes did not ↓ infraction ex vivo ↑ Contraction and relaxation ↓ End‐diastolic pressure ↑ pAkt, pGSK3 alpha/beta and ↓ pJnk ↓ Peripheral leukocytes	↑ ATP/ADP NADH/NAD+ ratios after reperfusion ↓ Protein oxidation	101
	Neonatal hyperoxic lung injury (rat)	Human UCB‐derived MSCs	↓ Apoptosis ↓ Pro‐inflammatory cytokines Enhanced effects with earlier MSC injections	↓ P47phox ↓ MPO	133
	O_2_‐induced bronchopulmonary dysplasia (rat)	Rat BM‐MSC‐CM	↓ Pulmonary hypertension ↓ Right ventricular hypertrophy ↓ Pulmonary arterial medial wall thickness ↓ Gross morphological damage to alveoli ↑ Therapeutic value from the CM of hyperoxic MCSs compared to normoxic MSCs Lung fibroblast CM had no therapeutic value	↑ hydroxyl radical antioxidant capacity in MSCs compared with lung fibroblast	61
	Renal I/R injury (rat)	Rat BM‐MSCs	↓ Cellular degeneration (histopathology) ↑ EGF MSCs had no effect on pERK1/2 MSCs had no effect on Bax and Bcl‐2	↑ HO‐1 ↓ DNA oxidation	93
	Renal I/R injury (rat)	Human WJ‐MSC micro vesicles	↑ Cell proliferation ↓ Apoptosis ↓ Fibrosis Improved kidney function	↓ ROS, lipid peroxidation and protein oxidation ↓ NOX2	134
	Semaxanib/hypoxia‐induced pulmonary arterial hypertension (rat)	Human BM‐MSCs	↓Hypertension In pulmonary artery: ↑ Metabolites TCA cycle‐associated metabolites ↓ Fructose and sorbitol—glycolysis associated	↑ GSH/GSSG and Cys/CySS ratios	87
	Small bowel I/R injury (rat)	Rat AT‐MSCs	↓ Intestinal permeability ↓ TNFα and NFκB ↓ Protein oxidation and lipid peroxidation ↓ Cytosolic cytochrome *c*, c‐caspase 3, c‐caspase 9, c‐PARP, APAF‐1 and mitochondrial Bax ↓ Cell proliferation ↓ Apoptosis ↓ Immune cells	↓ MPO and iNOS ↓ NOX1 and NOX2 ↑ NQO1, glutathione reductase, GPx1 ↑ HO‐1 cells	84
Tissue/cell engraftment	Ovarian tissue autograft (mouse)	Mouse AT‐MSCs	↑ Graft efficacy ↑ IL‐10 ↓TNFα and IL‐6 ↓ Apoptosis	↑ SOD activity ↓ Lipid peroxidation	70
Traumatic injuries	Spinal cord injury (canine)	Canine AT‐MSCs	↑ Motor function ↓ Hemorrhagic area ↓ Microglia ↓ TNFα, IL‐6 and COX2	↓ Lipid peroxidation and protein oxidation	42
	Testicular torsion injury (rat)	Rat AT‐MSCs	↓ Apoptosis	↓ Lipid peroxidation	135

Abbreviations: ALT, alanine aminotransferase; APAF‐1, apoptotic protease activating factor 1; AST, aspartate aminotransferase; AT‐MSC, adipose tissue‐derived MSC; BAX, Bcl‐2‐associated X protein; Bcl‐2, B‐cell lymphoma 2; BDNF, brain‐derived neurotrophic factor; bFGF, basic fibroblast growth factor; BM‐MSCs, bone marrow‐derived MSC; CAT, catalase; c‐caspase 3, cleaved‐caspase 3; CM, conditioned medium; CO, carbon monoxide; COX2, cyclooxygenase‐2; c‐PARP, cleaved poly (ADP‐ribose) polymerase; Cys, cysteine; DRG, dorsal root ganglion; DSS, dextran sulfate sodium; EGF, epidermal growth factor; ER stress, endoplasmic reticulum stress; GABA, gamma‐aminobutyric acid; GDNF, glial cell‐derived neurotrophic factor; GFAP, glial fibrillary acidic protein; GLUT1, glucose transporter 1; GPx, glutathione peroxidase; GSH, glutathione; H_2_O_2_, hydrogen peroxide; HO‐1, heme oxygenase‐1; I/R, Ischemia / reperfusion; ICAM, Intercellular adhesion molecule; IFNγ, interferon gamma; IL, interleukin; iNOS, inducible nitric oxide synthase; IV, intravenous; LPS, lipopolysaccharide; MAP2, microtubule associated protein‐2; MPO, myeloperoxidase; NAD(P)H, nicotinamide adenine dinucleotide phosphate hydrogen; ND, No difference; NFκB, Nuclear factor κB; NGF, Nerve growth factor; nNOS, neuronal nitric oxide synthase; NO, nitric oxide; NOX, NAD(P)H oxidase; NQO1, NAD(P)H quinone dehydrogenase 1; NRF2, nuclear factor erythroid 2‐related factor 2; NT3 and 4, neurotrophin 3 and 4; O2−, superoxide; ONOO−, peroxynitrite; p38MAPK, p38 mitogen‐activated protein kinases; pAkt, phosphorylated protein kinase B; PCNA, proliferating cell nuclear antigen; pGSK3, phosphorylated glycogen synthase kinase 3 beta; pJnk, phosphorylated c‐Jun N‐terminal kinases; pERK1/2, phosphorylated extracellular signal‐regulated kinases 1/2; RAGE, receptor for advanced glycation end products; ROS, reactive oxygen species; SOD, superoxide dismutase; STC‐1, stanniocalcin‐1; STZ, streptozocin; TCA cycle, tricarboxylic acid cycle; TGFβ, transforming growth factor beta; TNFα, tumor necrosis factor alpha; TSG6, TNFα‐stimulated gene‐6; TSP1, thrombospondin 1; Tuj1, neuron‐specific class III beta‐tubulin; UC‐MSC, umbilical cord‐derived MSC; αSMA, alpha‐smooth muscle actin; γ‐GCS, gamma‐glutamylcysteine synthetase; γ‐H2AX, gamma‐H2A histone family member X; Δψm, mitochondrial membrane potential; CySS, cystine (disulfide form of cysteine); GSSG, glutathione disulfide.

**TABLE 2:** Antioxidant effects of MSCs in in vitro models

Cell types	Model	MSCs used	Antioxidant and other effects of MSCs	References	
Cardiomyocytes and endothelial cells	Glucose‐deprived hypoxia‐reoxygenated H9c2 cardiomyocytes (rat)	Rat BM‐MSCs Direct coculture with GFP+ MSCs	↓ Apoptosis ↓ Bax ↑ Bcl‐2 ↓ Caspase 3 ↓ Δψm MSCs transferred mitochondria to H9c2 via TNT structures Inhibition of TNT formation partially reversed these effects	121	
	H_2_O_2_‐treated RL14 cardiomyocytes and human umbilical vein endothelial cells (HUVEC)	Human AT‐MSCs	MSCs engulf mitochondria from H_2_O_2_‐treated cells MSC coculture prevented cell death—no paracrine effect MSCs donate functional mitochondria to somatic cells exposed to H_2_O_2_ MSCs degrade engulfed mitochondria via autophagosomes MSCs do not prevent somatic cell death when mitophagy is inhibited Mitochondria sensing by MSCs ↑HO‐1 in MSC HO‐1 stimulated mitochondrial biogenesis in MSC which was necessary to prevent somatic cell death Doxorubicin caused increased mitochondrial O_2_^.−^ production and MSCs protected cells via a similar mechanism dependent on ROS generation and transfer of mitochondria from somatic cells	53	
	I/R of ventricular myocytes (mouse) in vitro	Mouse BM‐MSC‐CM	↓ Cell loss ↓ Early afterdepolarization of myocytes ↓ Excessive depolarization of Δψm after reperfusion ↓ Exaggerated hyperpolarization of Δψm after *acute* reperfusion—effect prevented by PI3K, Akt, and I_k,ATP_ inhibition I_k,ATP_ opener mimicked effects: ↓ Δψm hyperpolarization ↓ Mitochondrial O_2_^.−^ ROS scavenger mimicked effects: ↓cell loss, ↓early after depolarizations, ↓ Δψm hyperpolarization, ↓ O_2_^.−^	54	
	Oxygen glucose deprivation and reoxygenation of human umbilical vein endothelial cells (HUVEC)	Human BM‐MSCs	MSCs and HUVEC cells form tunneling nanotubes during oxygen glucose deprivation and reoxygenation Exchange of mitochondria in HUVECs and MSCs confirmed by mtDNA and fluorescent dye ↓ Cell death ↑ Oxygen consumption rate and ↓ extracellular acidification rate No effect by mitochondria‐depleted MSCs	136	
	Cytarabine‐treated human umbilical cord vein endothelial cells (HUVEC)	Human BM‐MSCs	Tunneling nanotubes facilitate bidirectional mitochondria transfer between MSCs and endothelial cells Unidirectional mitochondria donation to endothelial cells pretreated with cytarabine ↓ Apoptosis ↑ Capillary formation	122	
	*tert‐Butyl* hydroperoxide‐treated umbilical endothelial cells (human)	Human placental MSC‐CM	↓ ROS ↓ Apoptosis No effect on SOD1, CAT and GPx1 mRNA ↑ SOD2 mRNA and protein SOD2 expression correlated with IL‐6‐ST (gp130)‐STAT3 signaling SOD2 and STAT3 siRNA in endothelial cells reduced protective effects of MSC‐CM	77	
Fibroblasts	*tert‐Butyl* hydroperoxide‐treated human dermal fibroblasts	Human AT‐MSC‐CM	↑ Antioxidant capacity over normal culture media ↑ Cell survival ↓ Morphological damage ↑ SOD activity in human dermal fibroblasts ↑ GPx activity in human dermal fibroblasts	60	
	UV‐exposed fibroblasts (human)	Human UC‐MSC‐CM	↑ Cell viability ↑ SOD activity	73	
Glial cells and neurons	Activated microglia and NO‐induced neuronal death (rat)	Human BM‐MSCs	↓ Neuronal loss from activated microglia ↓ Neuronal loss from NO Neuroprotection prevented by SOD3 inhibition	71
	Amyloid‐β oligomer‐induced damage to hippocampal neurons (rat)	Rat BM‐MSCs Transwell coculture and exosomes	↓ ROS MSCs internalize amyloid‐β oligomers Exosomes exhibit catalase activity Inhibition of CAT abrogates effect of exosomes Amyloid‐β does not affect MSC viability, proliferation, or cellular respiration	83
	Glucose‐deprived hypoxia‐reoxygenated primary astrocytes (human)	Human dental pulp‐derived and BM‐MSCs Transwell and CM	↑ Viability of astrocytes ↓ ROS ↓ IL‐1β after MSC‐CM ↓ Astrogliosis	137
	Glucose‐deprived scratch injured T98G glioblastoma cells (human)	Human AT‐MSC‐CM	↑ Wound closure ↑ Viability ↓ ROS	138
	H_2_O_2_‐treated cortex‐derived neural stem cells (rat)	Rat BM‐MSC‐CM	↓ Apoptosis ↓ Lipid peroxidation ↑ SOD activity	74
	H_2_O_2−_treated motor neurons (NSC‐34) expressing human mutant SOD1 (ALS)	Mouse AT‐MSC exosomes	↑ Cell viability of naïve cells and SOD1 mutant cells	139
	H_2_O_2_‐treated retinal ganglion cells (RGCs) (rat)	Rat BM‐MSCs Transwell	↓ Apoptosis ↓ Lipid peroxidation and ↑SOD activity in RGCs ↓ IL‐1β and TNFα in supernatant ↑ BDNF and CNTF in RGCs	75
	H_2_O_2_‐treated SH‐SY5Y neuroblastoma cells (human)	Human AT‐MSC CM	↑ Viability ↓ Antioxidant capacity ↓ ROS Restored electrophysiological properties Effects replicated by NAC	140
	Sevoflurane‐induced apoptosis in human neuroglioma H4 cells	Rat BM‐MSCs	Transwell cultures ↓ c‐caspase 3 and Bax ↓ ROS ↓ Cytochrome *c* translocation ↑ ATP	141
Hepatocytes	Acetaminophen and H_2_O_2_‐treated human hepatocytes (HepG2)	Rat BM‐MSC‐CM	Exosome‐rich fractionated conditioned medium ↑ Cell viability ↓ ROS	142
	H_2_O_2_‐treated AML12 hepatocytes (murine)	Mouse BM‐MSC extracellular vesicles	↓ ROS ↓ Pro‐inflammatory cytokines	143
	H_2_O_2_‐treated human fetal hepatocytes (LO2 cells)	Human UC‐MSCs extracellular vesicles	↓ ROS ↓ Mitochondrial O_2_^.−^ ↓ Apoptosis Exosomes contain PRDX1‐6, SOD1‐2, CAT, TXN GSTO and GSTP1 Silencing of SOD2 in MSCs inhibits therapeutic effect of exosomes	80
Immune cells	Cytarabine or methotrexate‐treated immortalized human T lymphocytes (Jurkat cells)	Human BM‐MSCs	Jurkat cells transfer mitochondria to MSCs after exposure to chemotherapeutics. Few mitochondria transferred from MSCs to Jurkat cells MSC direct coculture: ↓ Apoptosis ↓ Mitochondrial O_2_^.−^ Effects blocked by inhibition of mitochondrial transfer using cytochalasin D and anti‐ICAM1	120
	LPS‐stimulated blood‐derived monocytes (human)	Human AT‐MSCs	↓ TNFα ↓ Nitrite ↓ COX2 ↓ MPO ↓ ROS	110
	LPS‐treated human monocyte‐derived macrophages	Human BM‐MSC CM and extracellular vesicles	↑ oxygen consumption rate ↑ phagocytic phenotype This effect was partially reversed by Ab blocking extracellular vesicles (anti‐CD44) Extracellular vesicles from MSCs transfer mitochondria to macrophages MSC‐CM ↑ M2 phenotype (anti‐inflammatory) Effects abolished by damaging mitochondria in MSCs	114
	LPS‐treated neutrophils (human)	Human UC‐MSCs Transwell and extracellular vesicles	↓ Lipid peroxidation ↓ ROS ↓ MPO activity No effect on cell numbers in vitro	80
	Macrophages in vitro (human and mouse)	Human BM‐MSCs	↓ ROS‐associated with NRLP3 inflammasome activation ↓ NRLP3 associated caspase 1 activation ↓ NRLP3 associated IL‐1β and IL‐18 secretion ↓ TNFα and IL‐6 transcription Effects inhibited by STC‐1 siRNA	111
	PMA‐activated neutrophils (mouse and human)	Human AT‐MSCs	↓ Respiratory burst (ROS) dependent on SOD3 expression by MSCs ↓ Apoptosis ↓ MPO protein and activity	79
Islet cells	Cytokine cocktail‐exposed islet cells (rat)	Human BM‐MSCs	IL‐1, TNFα and IFNγ cocktail. ↑ Insulin secretion ↑ SOD1, ↑ NQO1, ↑HO‐1 ↑Ferritin H	78
	Hypoxia (1% O_2_) exposed porcine islet cells	Human UC‐MSC CM and exosomes	↓ Apoptosis ↓ ROS ↓ Mitochondrial O_2_^. −^ ↑ GSH, ↑ GPx activity Inhibition of ERK pathway reversed effects MSCs secreted high levels of IL‐6 MSC exosomes and recombinant IL‐6 ↓apoptosis and ↓ROS	144
	Hypoxia‐exposed neonatal porcine islet cell clusters (porcine)	Human UC‐MSC CM and exosomes	↓ Apoptosis ↑ Oxygen consumption rate Effects reduced after clearance of exosomes in conditioned media	113
	Normoxia‐ and hypoxia‐exposed WJ‐MSC engineered islet‐like cells (human)	Human WJ‐MSCs	Normoxia (21% O_2_) and hypoxia (2% O_2_) WJ‐MSCS formed monolayer while islet‐like cells were free floating ↑ Proliferation ↓apoptosis and ↓ROS in both conditions ↓ NO and O_2_^.−^ in hypoxia	145
	Primary islet cells (mouse)	Mouse BM‐MSCs Transwell coculture	↑ GSTM1	91
Keratinocytes	High glucose and LPS‐treated primary keratinocytes (rat)	Rat BM‐MSC‐CM	↑ Viability ↑ Wound assay closure ↓ ROS Dependent on ERK signaling	146
Lung epithelial cells	H_2_O_2_‐treated human alveolar basal epithelial adenocarcinoma cells (A549)	Human BM‐MSCs	↑ Cell viability ↑ Transcription and protein expression of STC‐1 in H_2_O_2_‐treated MSCs ↓ Cell viability with Anti‐STC‐1 ↑ Cell viability with recombinant STC‐1 Similar results in H1299 and PC9 ↓ Cell viability with STC‐1 siRNA MSCs ↑ ROS with STC‐1 siRNA MSCs ↑ mRNA expression of uncoupling protein 2 in A549 ↓ mRNA expression of uncoupling protein 2 with anti‐STC‐1	100
Osteocytes	Mitochondrial DNA (mtDNA)‐depleted 143B osteosarcoma cells (human)	Human WJ‐MSCs	MSCs in direct co culture donated mitochondria MSCs and mitochondria‐depleted cells removed via auxotrophic restriction Recovered cellular respiration (oxidative phosphorylation) Restoration of cellular proliferation and motility Effects of mitochondria donation sustained for 45 passages	124
Renal cells	Cisplatin‐treated renal proximal tubular cells (rat)	Human UC‐MSC exosomes	↓ Δψm ↑ PCNA ↓ Oxidized DNA ↓ Lipid peroxidation ↑ GSH ↓ Bax, ↑ Bcl‐2	27
	H_2_O_2_‐treated renal tubular epithelial cells (rat) in vitro	Rat BM‐MSCs	↓ Apoptosis ↓ Cell loss ↑ Mitosis ↓ Bax expression ↑ p‐ERK1/2	93
	High glucose‐treated glomerular mesangial cells (rat)	Rat BM‐MSC‐CM	↓ ROS ↓ GLUT1 Inhibition of HGF via antibody blocking inhibited antioxidant effect	29
	Hypoxia reoxygenation of rat kidney epithelial cells (NRK‐52E)	Human WJ‐MSC extracellular vesicles	↓ ROS ↑ Activated NRF2 ↑ ARE activity ↑ HO‐1	52
	Oxalate and calcium oxalate monohydrate‐treated human proximal tubular epithelial (HK‐2)	Human UC‐MSC exosomes	↓ Apoptosis ↓ Lipid peroxidation ↓ H_2_O_2_ ↓ ROS ↓ LDH ↓ Mesenchymal markers ↓ Migration	147
Skeletal muscle cells	Dexamethasone‐induced muscle atrophy in L6 rat skeletal muscle cells	Human UC‐MSC‐CM	↑ Muscle related gene expression (myogenin, desmin) ↑ SOD activity ↓ ROS generation ↑ CAT, SOD1, GPx‐1 in L6 cells	76
	Dexamethasone‐induced muscle atrophy in L6 rat skeletal muscle cells	Human UC‐MSC (isolated mitochondria) Centrifugal delivery of exogenous mitochondria	↑ Cell proliferation ↑ Δψm ↑ ATP content ↓ Mitochondrial O_2_^.−^	125
Trophoblasts	Hypoxia (1% O_2_) trophoblast cells (mouse)	Mouse BM‐MSCs Transwell	↑ Mitofusin‐2 ↑ β‐HCG and progesterone ↑ ATP levels ↓ Caspase 3 and 9 ↓ Bax, ↑Bcl‐2 ↓ Apoptosis	148

Abbreviations: ARE, antioxidant response element; AT‐MSC, adipose tissue‐derived MSC; BAX, Bcl‐2‐associated X protein; BDNF, brain‐derived neurotrophic factor; BM‐MSCs, bone marrow‐derived MSC; c‐caspase 3, cleaved‐caspase 3; CM, conditioned medium; CNTF, ciliary neurotrophic factor; COX2, cyclooxygenase‐2; ERK, extracellular signal‐regulated kinases; GLUT1, glucose transporter 1; GPx, glutathione peroxidase; GST, glutathione S‐transferase; H_2_O_2_, hydrogen peroxide; HGF, hepatocyte growth factor; HO‐1, heme oxygenase‐1; I/R, ischemia/reperfusion; ICAM, intercellular adhesion molecule; IFNγ, interferon gamma; IL, interleukin; LDH, lactate dehydrogenase; LPS, lipopolysaccharide; M2, type‐2 macrophages; MPO, myeloperoxidase; NAC, N‐acetyl cysteine; NAD(P)H, nicotinamide adenine dinucleotide phosphate hydrogen; NO, Nitric oxide; NQO1, NAD(P)H quinone dehydrogenase 1; NRF2, nuclear factor erythroid 2‐related factor 2; NRLP3, nod‐like receptor protein‐3; O_2_^.−^, superoxide; pERK1/2, phosphorylated extracellular signal‐regulated kinases 1/2; PRDX1‐6, peroxiredoxin; RGCs, retinal ganglion cell; ROS, reactive oxygen species; SOD, superoxide dismutase; STAT3, signal transducer and activator of transcription 3; STC‐1, stanniocalcin‐1; TNFα, tumor necrosis factor alpha; TNT, tunneling nanotube; TXN, thioredoxin; UC‐MSC, umbilical cord‐derived MSC; β‐HCG, β‐human chorionic gonadotropin; Δψm, mitochondrial membrane potential.

Oxidative stress refers to a deviation from the physiological redox state and an increase in pro‐oxidants, or free radicals, that structurally change lipids, proteins, and DNA in a way that causes pathology or damage to a cell or tissue.^6^ The most widely studied free radicals are reactive oxygen species (ROS), which can also include reactive molecules that have a stable charge. The three major endogenous ROS include the superoxide anion (O_2_^.−^), hydroxyl radical (•OH), and hydrogen peroxide (H_2_O_2_).^7^, ^8^ O_2_^.−^ is predominantly generated by nicotinamide adenine dinucleotide phosphate reduced (NADPH)‐oxidase (NOX) family enzymes or, by the mitochondria, as a by‐product of oxidative phosphorylation.^9^ The level of mitochondria‐derived O_2_^.−^ depends on metabolic substrates, cytosolic Ca^2+^ levels, pH, and oxygen tension.^10^ O_2_^.−^ generated from complexes of the electron transport chain (ETC) are highly reactive and can damage the mitochondrion.^11^ The detoxification of O_2_^.−^ into H_2_O_2_ is mediated by superoxide dismutase (SOD).^9^ However, H_2_O_2_ can also be generated in various metabolic processes and by dual oxidases (DUOX).^12^ While H_2_O_2_ is more stable than O_2_^.−^, its detoxification is crucial as it possesses a weak peroxide bond that makes it susceptible to reacting with metals, such as Fe^2+^, to generate reactive •OH through the Fenton reaction.^13^ Both, H_2_O_2_ and O_2_^.−^, are diffusible across cell membranes and can promote cell death and inflammatory signaling.^14^, ^15^ Several studies have demonstrated that MSCs can reduce ROS and biomarkers of oxidative stress. In this review, evidence of direct and indirect antioxidant mechanisms of MSC therapies is explored.

## 2 MSCs ARE RESISTANT AND RESPOND TO OXIDATIVE STRESS

The therapeutic properties of MSCs have been explored in many models of disease associated with high levels of ROS and biomarkers of oxidative injury. MSCs must survive these volatile environments to exert their therapeutic effects, which can present as a challenge for their engraftment after administration. Nonetheless, several studies have demonstrated that MSCs are highly resistant to oxidative insult. The oxidative effects of ionizing radiation are limited on MSCs which have been attributed to their ability to directly scavenge free radicals.^16^ It has been demonstrated that MSCs are resistant to oxidative and nitrosative stimuli in vitro which is associated with constitutively expressed antioxidant enzymes SOD1, SOD2, catalase (CAT), and glutathione peroxidase (GPx), in addition to high levels of the antioxidant glutathione (GSH).^17^ Depletion of GSH results in a loss of tolerance to oxidative stress. MSCs also constitutively express heat‐shock protein 70 (HSP70) and sirtuin (SIRT)3,^18^ which may also play a role in the resistance of MSCs to oxidative/nitrosative injury. SIRT1 is also required for MSC survival against H_2_O_2_ and its overexpression has a protective effect.^19^ Likewise, SIRT6 has been suggested to confer resistance to oxidative insult and basal ROS production in MSCs via downstream production of antioxidants including heme oxygenase‐1 (HO‐1).^20^ Overexpression of HO‐1 ameliorates elevations in ROS and cellular senescence in SIRT6‐null MSCs and, therefore, appears to be a critical component of the survival mechanism of MSCs in oxidative environment.^20^

In addition to wielding constitutive antioxidants, MSCs are also capable of significant adaptions in response to redox stress. MSCs exposed to lipopolysaccharide (LPS) produce oxidative and nitrosative free radicals.^18^ In parallel, several adaptive processes are observed including the upregulation and/or nuclear translocation of redox‐sensitive factors (nuclear factor kappa‐B [NFκB], thioredoxin [TRX1], apurinic/apyrimidinic endonuclease redox effector factor‐1 [APE1/Ref‐1], nuclear factor erythroid 2‐related factor 2 [NRF2], forkhead box O3 [FOXO3]. and HO‐1), as well as mitochondrial remodeling and autophagy. Similarly, MSCs exposed to hypoxic conditions (1.5%‐2% O_2_) exhibit increased intracellular ROS and cells respond by upregulating the expression of hypoxia‐inducible factor 1 alpha (HIF‐1α), erythropoietin receptor, CAT, HO‐1, and antiapoptotic Bcl‐2 family of proteins.^21^ Exposure of MSCs to hypoxia during in vitro culture can enhance their anti‐inflammatory, antioxidative, and cytoprotective properties.^21^, ^22^, ^23^ This suggests that the ability of MSCs to tolerate and respond to oxidative environment may be critical to their engraftment and therapeutic efficacy at sites of tissue injury.

## 3 EFFECT OF MSCs ON OXIDATIVE STRESS BIOMARKERS AND ROS

In disease models, oxidative stress is typically quantified via biomarkers of oxidation to DNA and proteins or lipid peroxidation. Administration of MSCs has been demonstrated to reduce levels of one or more of these markers in a variety of animal models associated with oxidative stress (Table 1). Injection of MSCs themselves may not be critical to their antioxidant effects as administration of their conditioned medium (CM) also reduced lipid peroxidation in a model of ureteral obstruction‐induced kidney injury.^24^ Administration of MSC‐derived exosomes was also effective to rescue protein oxidation and lipid peroxidation in animal models of septic and hyperglycemic brain injury and cognitive impairment.^25^, ^26^ Likewise, DNA oxidation and lipid peroxidation caused by cisplatin‐induced kidney damage are alleviated by exosomes from human umbilical cord‐derived MSCs (UC‐MSCs); these results were confirmed in vitro with renal proximal tubular cells.^27^ Treatments with MSCs were also demonstrated to reduce levels of ROS in animal models of diabetic retinopathy and nephropathy, severe acute pancreatitis, ureteral obstruction‐induced kidney damage, Alzheimer's disease, and metabolic renovascular disease.^24^, ^28^, ^29^, ^30^, ^31^, ^32^ Specifically, MSC treatments have been shown to reduce levels of H_2_O_2_ in intestinal inflammation and several organs in a model of premature aging.^33^, ^34^ MSCs also reduced levels of O_2_^.−^ in colitis and spontaneous stroke.^34^, ^35^

Typically, the reduction of oxidative stress markers by MSC treatments is associated with functional recovery and positive outcomes in animal models. The exception to this is the diversity of responses at various stages of hepatocarcinoma whereby antioxidant effects of MSCs reduce tumor burden at the early stages of disease by protecting the integrity of DNA but increase tumor progression at the late stages of the disease possibly by reducing ROS‐associated cell death.^36^ The timing of treatments may also affect MSCs ability to attenuate oxidative stress as pretreatment with MSCs is more effective to prevent oxidative stress in septic lung injury and acute liver failure.^37^, ^38^

Several studies have also investigated the therapeutic properties of MSCs on nitrosative stress which is particularly of interest in neural diseases. MSCs reduced the volatile peroxynitrite (ONOO−) in a model of intracerebral hemorrhage and nitrite levels in diabetic sensory neuropathy.^39^, ^40^ In a model of radiation‐induced neurological complications, intranasal delivery of MSCs reduced inducible nitric oxide synthase (iNOS) expression and oxidative stress biomarkers, which are associated with improved cognitive performance and neuronal survival.^41^ In a model of spinal cord injury, adipose tissue‐derived MSCs (AT‐MSCs) also demonstrated antioxidant activity with a reduction in lipid peroxidation and protein oxidation; however, no significant effects were observed for nitrosylation.^42^ Furthermore, MSCs have been associated with increased nitric oxide (NO) in a model of Alzheimer's disease which may have been driven by preventing the loss of neuronal nitric oxide synthase (nNOS).^43^ Alternatively, several studies observed a reduction in inflammation‐induced iNOS and nNOS expression after MSC treatment.^37^, ^44^, ^45^, ^46^, ^47^ Overall, while the majority of studies support antioxidative effects of MSCs, their antinitrosative effects are unclear and likely to be disease and tissue specific. Furthermore, NO can be produced by nonhuman MSCs which is thought to be critical to their immunomodulatory function which may also explain these inconsistent effects on nitrosative stress.^42^, ^48^

Although studies in cells and animal models unequivocally demonstrate that MSC treatments reduce levels of oxidative stress, albeit limited data exist from human studies. Nonetheless, favorable outcomes in a case study utilizing MSCs to treat the lungs of a subject previously exposed to sulfur mustard gas were attributed to the antioxidant properties of MSCs as evidenced by reduced lipid peroxidation levels in the sputum.^49^

The antioxidant effects of MSC treatments are likely to be a specific property of these cells as they are more efficacious than hematopoietic stem cells and fibroblast at reducing oxidative stress in carbon tetrachloride‐induced‐liver injury and sepsis, respectively.^50^, ^51^ Likewise, fibroblast exosomes have no effect on kidney injury‐induced by ischemia and chemotherapy.^27^, ^52^

The alleviation of oxidative stress in animal models is associated with decreased pro‐inflammatory cytokines and markers of cellular death highlighting the close association between these processes. The precise mechanisms of in vivo MSC treatments are difficult to determine as cell death, inflammation, and oxidative stress occur concomitantly and perpetuate each other. However, a growing body of evidence suggests that MSCs have a direct role on suppressing oxidative stress and ROS production which may mediate their antiapoptotic and anti‐inflammatory effects.

## 4 ANTIOXIDATIVE MECHANISMS OF MSCs

The potential for MSCs to attenuate oxidative injury is unequivocally demonstrated by the reduction in ROS and biomarkers of oxidative stress in many disease models. Evidence form in vitro models suggests that MSCs directly protect cells from oxidative stimuli (Table 2). This is often associated with a reduction in ROS suggesting that MSCs avert the negative effects of oxidative stress by reducing the oxidative stimuli. The antioxidative effects of MSCs often occur in a paracrine manner in vitro and the administration of MSC‐conditioned medium (CM) can also reduce oxidative stress in vivo suggesting a paracrine component to their mechanism.^24^ Nevertheless, others report that the antioxidant effects of MSCs can be cell contact dependent^53^; albeit, these mechanisms could be disease and tissue dependent. Currently, MSCs have been proposed to reduce oxidative injury via scavenging free radicals, enhancing host antioxidant defenses, modulating the inflammatory response, augmenting cellular respiration and mitochondrial functions, or donating their mitochondria to protect damaged cells (Figure 1).^37^, ^50^, ^51^, ^53^, ^54^

### 4.1 Antioxidant defense and scavenging

To maintain redox homeostasis and prevent excessive production of free radicals, cells rely on a complement of enzymatic antioxidants, including SODs, CAT, GPx, and small nonenzymatic antioxidants, such as GSH. After MSC treatments, the total antioxidant capacity of tissues are enhanced as observed in models of chemotherapy‐induced neuropathy, hyperglycemia‐induced cardiac damage, systemic sclerosis, hepatocarcinoma, and acute liver injury.^36^, ^55^, ^56^, ^57^, ^58^ MSCs are receptive to oxidative stimuli and exhibit all necessary machinery to efficiently process ROS.^18^, ^59^ Furthermore, media conditioned by MSCs have potent antioxidant capacity indicating that MSCs actively secrete antioxidants.^60^ MSC‐CM has more effective antioxidant properties than CM from lung fibroblasts.^61^ In several models of disease, MSC treatments upregulate the expression of antioxidant defense enzymes in vivo (Table 1). Therefore, the antioxidant effects of MSCs may be explained by their ability to directly scavenge free radicals and by enhancing antioxidant defenses in host tissues through upregulation of antioxidant enzymes.

Volatile O_2_^−^ is produced during cellular respiration by the mitochondria and NOX enzymes during tissue inflammation; O_2_^−^ is eliminated by SOD which catalyzes its conversion to H_2_O_2_. Antioxidant effects of MSC treatments have been associated with enhanced SOD activity or the expression of SODs in models of aging, age‐related erectile dysfunction, chemotherapy‐induced pulmonary fibrosis or gonadotoxicity colitis, pancreatitis, septic lung injury, arthritis, hepatotoxicity and hepatic ischemia reperfusion injury, Alzheimer's disease, and ovarian autografts.^33^, ^34^, ^37^, ^38^, ^43^, ^50^, ^62^, ^63^, ^64^, ^65^, ^66^, ^67^, ^68^, ^69^, ^70^ MSCs secrete all isoforms of SOD including SOD1 and SOD2, which are archetypically not released extracellularly.^33^, ^59^, ^71^, ^72^ Thus, it is difficult to interpret whether enhanced SOD activity and/or SOD expression is due to MSCs or host‐tissue‐derived SOD. In in vitro studies, MSC‐CM or MSCs in transwell cocultures promote SOD activity in *tert‐Butyl* hydroperoxide or UV‐exposed fibroblasts, H_2_O_2_‐treated neural stem cells or retinal ganglion cells, and dexamethasone‐induced muscle atrophy model.^60^, ^73^, ^74^, ^75^, ^76^ These studies suggest that MSCs enhance SOD activity in cells exposed to oxidative stimuli. Likewise, MSC‐CM increases SOD1 expression in islet cells exposed to pro‐inflammatory cytokines and SOD2 in *tert‐Butyl* hydroperoxide‐treated umbilical endothelial cells.^77^, ^78^ In endothelial cells, increased SOD2 expression was regulated by signal transducer and activator of transcription (STAT3) signaling and knockdown of either SOD2 or STAT3 decreased the antiapoptotic effects of the MSC‐CM.^77^ These findings suggest that MSC upregulation of SOD in host tissues may be critical to their antioxidant effects. Alternatively, MSCs stimulated with TNF‐α and IFN‐γ were found to secrete high levels of SOD3, which was a major contributor to the antioxidant properties of MSCs in the amelioration of NO‐induced neuronal death in vitro.^71^ Likewise, SOD3 expression in MSCs was necessary to suppress neutrophil respiratory burst and the accumulation in immune complex‐mediated dermal vasculitis.^79^ Similarly, silencing of SOD2 in MSCs inhibits the antioxidant properties and therapeutic efficacy of their exosomes in hepatic I/R injury in vivo and H_2_O_2_‐treated human fetal hepatocytes which can be recovered by the addition of a SOD2 mimetic.^80^

CAT and GPx are responsible for detoxifying H_2_O_2_ by its conversion to oxygen and water. MSCs secrete CAT and upregulation of CAT expression is associated with the therapeutic properties of MSCS in models of Friedreich's ataxia, radiation‐induced aortic injury, septic lung injury, and hepatoxicity.^33^, ^37^, ^50^, ^68^, ^81^, ^82^ Likewise, upregulation of CAT associates with the antioxidant properties of MSCs in aging and colitis which parallels measurable reductions in H_2_O_2._ ^33^, ^34^ Exosomes derived from MSCs also express functional CAT.^31^, ^83^ Inhibition of CAT suppresses their protective effects on amyloid‐β oligomer‐induced damage to hippocampal neurons indicating that this enzyme may also mediate antioxidant effects of MSCs.^83^ MSC treatments have also been demonstrated to upregulate the expression of GPx in septic lung injury, severe acute pancreatitis, small bowel ischemia/reperfusion (I/R) injury, and Friedreich's ataxia.^37^, ^67^, ^81^, ^84^ MSC‐CM can also enhance GPx activity in fibroblasts under oxidative stimuli.^60^ Therefore, both CAT and GPx may play a role in MSC treatments in reducing oxidative stress by the elimination of H_2_O_2_ which was commonly used as a cell death inducing oxidative stimulus in in vitro studies.

The glutathione system is critical to scavenging ROS in animals, plants and fungi. GSH exerts its antioxidant effects by reducing free radicals and peroxides. During this process, glutathione disulfide (GSSG) is generated which is converted back to GSH by NADPH and is catalyzed by glutathione reductase. Increased levels of GSSG to GSH are suggestive of an oxidative redox state. Conversely, higher GSH‐to‐GSSG ratio is observed after MSC treatment in models of type 2 diabetes, in the hippocampus after chronic ethanol intake and hypoxia‐induced hypertension.^85^, ^86^, ^87^ Likewise, MSCs improve GSH‐to‐GSSG ratio in sepsis which is not achieved by fibroblast.^51^ Similarly, MSCs were found to increase GSH levels in diabetic models, septic lung injury, arthritis, hepatotoxicity, and epilepsy.^37^, ^38^, ^66^, ^68^, ^88^, ^89^, ^90^ These effects were also observed by MSC‐CM in a model of unilateral ureteral obstruction which may suggest GSH was host‐tissue‐derived.^24^ The effects of MSCs on GSH levels may be mediated by their ability to upregulate glutathione reductase which was observed in septic lung injury, acute pancreatitis, and I/R injury of the small bowel.^37^, ^67^, ^84^ Notably, MSCs have also been shown to upregulate the expression of glutathione S‐transferases (GSTs) in cells and tissues which detoxify many damaging molecules that arise from redox imbalance such as peroxidized lipids.^88^, ^91^ Glutathione reductase and GSTs are secreted by MSCs via exosomes.^31^ While GSH has been the best studied scavenger in MSC treatments, they have also been demonstrated to upregulate the expression of the enzyme NAD(P)H quinone oxidoreductase 1 (NQO1) which detoxify quinones that contribute to the generation of ROS in septic lung injury, small bowel I/R injury in vivo, and cytokine‐exposed islet cell in vitro.^78^, ^84^, ^92^

The multifunctional antioxidant HO‐1 has also been implicated in the therapeutic effects of MSC treatments. HO‐1's antioxidant and therapeutic mechanisms have been attributed to its ability to degrade heme, which is pro‐oxidative and the scavenging abilities of its products, biliverdin and bilirubin. HO‐1 expression is inducible in response to oxidative stress via nuclear factor erythroid 2‐related factor 2 (NRF2) which appears to be important in the adaptive responses of MSCs to inflammation and ROS.^18^ Downregulation of NRF2 and HO‐1 has been observed after MSC treatment of chemotherapy‐induced pulmonary fibrosis and diabetic sensory neuropathy which may be explained by a reduction in oxidative stimuli.^40^, ^63^ Conversely, the antioxidant effects of MSCs in several other models associate with upregulation of HO‐1 such as radiation‐induced aortic injury, septic lung injury, pancreatitis, and renal injuries caused by altered oxygen tensions and cisplatin.^30^, ^82^, ^92^, ^93^, ^94^ HO‐1 was determined to partially contribute to the effects of MSCs in pancreatitis as its inhibition with zinc protoporphyrin negated some of the effects of MSC treatments, including upregulation of CAT and increased SOD activity, which may indicate that these processes are downstream of HO‐1 activity.^30^ After MSC treatment of small bowel I/R injury, a larger number of HO‐1 expressing cells were observed which did not appear to completely colocalize with engrafted MSCs suggesting that treatments may increase HO‐1 expression in cells of the host.^84^ It has been demonstrated that overexpression of HO‐1 in MSCs enhances their therapeutic activity in septic lung injury which was attributed to its pro‐survival properties.^95^ Others have reported that MSCs still respond efficiently to oxidative insult with silenced HO‐1 by upregulating GSH pathway enzymes.^96^ Nevertheless, the CM of MSCs derived from HO‐1^−/−^ mice are unable to attenuate cisplatin‐induced renal injury and therefore HO‐1 appears to have an important role in the antioxidant properties of the MSC secretome.^94^

Together, these studies demonstrate that antioxidants secreted by MSCs and their ability to upregulate host antioxidant defenses contribute to the suppression of oxidative stress. The exosomes derived from MSCs appear to be particularly rich in machinery to process ROS and can include, but not limited to, GPx, GSTs, SOD1‐3, peroxiredoxin 1‐6, CAT, cytoglobin, prostaglandin‐endoperoxide synthase 1, peroxidasin, albumin, apolipoprotein E, glutathione‐disulfide reductase, and thioredoxin reductase 1‐2.^31^, ^80^ Notwithstanding, recombinant application of factors secreted by MSCs, such as hepatocyte growth factor (HGF) and basic fibroblast growth factors (bFGF), has been demonstrated to upregulate Gpx1, CAT, and SOD activity via SIRT1 and FOXO1 during age‐related loss of ovarian function.^97^ Mechanisms of antioxidant defense mediated by scavenging of ROS by MSCs and the host could occur independently or simultaneously and are likely to be disease‐specific.

### 4.2 Antioxidant effects on inflammation

Immune function is regulated by free radicals and the redox system; leukocytes and pro‐inflammatory mediators enhance the formation of free radicals and perturb the redox environment creating a positive feedback cycle.^98^ The immunomodulatory action of MSCs is a well‐documented phenomenon; however, their role in the interactions between the immune system and oxidative stress is not fully understood. Oxidative and/or nitrosative free radicals unequivocally play a role in all grades of acute and chronic inflammation. At physiological levels, they act as cellular signals modifying function and initiating necessary cell death programs. However, excessive generation of free radicals and/or inadequate scavenging results in protein oxidation, lipid peroxidation, and DNA damage that can be detrimental both intrinsically to the cell and the surrounding microenvironment. The ROS and reactive nitrogen species involved can take many forms and be generated from a variety of sources. Large amounts of the highly reactive O_2_^−^ anion are generated from NOX expressed by innate leukocytes.^99^ SOD catalyzes the conversion of O_2_^−^ to H_2_O_2_ which phagocytes and neutrophils use to generate hypochlorous acid (HOCl) via myeloperoxidase (MPO).^99^ Collectively, this is referred to as respiratory burst, a crucial element of the bactericidal response and inflammatory signaling. Nonetheless, MPO activity and O_2_^−^ are also associated with various inflammatory diseases. In inflammatory bouts, there is often a parallel increase in the expression of iNOS in leukocytes and, thus, subsequent generation of the free radical NO. Nonimmune cells such as epithelial cells are also capable of expressing iNOS and NOX to generate NO and O_2_^−^.

MSC treatments reduce inflammation and oxidative stress in colitis, pancreatitis, arthritis, sepsis, vasculitis, stroke, myocardial infarction, hyperoxic lung injury, and I/R injury of the kidneys and bowel (Table 1). These effects have included a reduction in inflammatory cytokines TNFα, IFNγ, interleukin (IL)‐1β, IL‐6, IL‐9, and IL‐4; decreased expression of ROS producing enzymes NOX, MPO, and iNOS; as well as a net reduction in the infiltration of immune cells such as neutrophils (Table 1). The anti‐inflammatory effects of MSCs in pancreatitis were partially dependent on their expression of the antioxidant pathway enzyme HO‐1.^30^ Previously, it was demonstrated in a model of sepsis that MSC treatments can reduce pro‐inflammatory cytokines in the serum and normalize thiol/disulfide redox pairings responsible for free radical scavenging.^51^ Decreased levels of IL‐1β and TNFα superseded restoration of redox homeostasis. This suggests that the aversion of oxidative injury was secondary to the immunomodulation of pro‐inflammatory signaling, at least in acute septic inflammation. Conversely, MSCs can directly reduce oxidative injury in many cell types in vitro; thus, it is likely that MSCs may also reduce oxidative stress in tissues by mechanism other than suppressing the immune system.^27^, ^54^, ^60^, ^75^, ^77^, ^100^ This is highlighted in in vivo and organotypic ex vivo models of myocardial I/R injury where MSC‐derived exosomes ameliorated infarction injury without altering leukocyte recruitment.^101^ In in vivo experiments, protein oxidation was reduced by MSC‐derived exosomes after 1 hour; neutrophils were yet to infiltrate into the tissue. After 24 hours, MSCs reduced peripheral blood leukocyte numbers and neutrophil infiltration into the myocardium; thus, the antioxidative activity of MSCs preceded signals recruiting leukocytes.^101^ This suggests that MSCs can attenuate oxidative stress‐induced tissue injury first, which can limit the recruitment of immune cells and subsequent inflammation in this model. This may be mediated by their ability to suppress NOX1 and 2 on resident cells which are downregulated by MSC treatments in acute myocardial infarction, sepsis‐induced brain injury, acute ischemic stroke, I/R injury to kidneys, and small bowel which were all associated with reduced inflammation.^25^, ^84^, ^102^, ^103^, ^104^

Neutrophils appear to be key mediators of oxidative stress in inflammation. These cells harbor an abundance of MPO, a major catalyst for hypochlorite and NO‐derived oxidants.^105^, ^106^ MSCs attenuate the infiltration of neutrophils and reduce MPO levels in several disease models.^50^, ^107^, ^108^ MSCs can also directly dampen the respiratory burst in neutrophils and suppress MPO activity required to produce free radical required for their pro‐inflammatory function which was dependent on SOD3 and occurs in a paracrine manner.^79^, ^80^, ^109^ Likewise, MSCs can also directly decrease ROS and MPO in stimulated monocytes and macrophages which suppress their pro‐inflammatory phenotype.^110^, ^111^ These data suggest that MSCs not only suppress the immune system to prevent oxidative injury, but also that their mechanism of immunosuppression is reliant on their antioxidant properties.

### 4.3 Cellular bioenergetics

Free radicals are produced by several metabolic processes and the mitochondria during cellular respiration. Dysfunction in mitochondria can cause cellular injury which is mediated through the generation of O_2_^.−^ and proteins that initiate cellular apoptosis. Depolarization of the mitochondrial membrane potential (Δψm) is a hallmark of mitochondrial dysfunction leading to cell death. Hyperglycemia can also cause oxidative stress via several mechanisms including the formation of free radical as by‐products of glucose auto‐oxidation that deplete antioxidant defense and advanced glycation end products that induce cellular stress. In models of hyperglycemia, MSC treatments can reduce the expression of glucose and fatty acid transports in kidney and cardiac tissue cells, which prevents glucose transport and ROS generation.^29^, ^56^ Therefore, the antioxidant effects of MSC treatments in models of diabetes may be downstream of glycemic control. Conversely, regulation of mitochondrial function and oxidative phosphorylation by MSCs has been implicated in several disease models. MSC treatments improve chemotherapy‐induced cognitive impairment, which associates with enhanced respiratory capacity of the mitochondria.^112^ The effects of MSCs on the mitochondria appear to occur in a paracrine manner as MSC‐CM increases the oxygen consumption rate of hypoxia‐exposed neonatal porcine islet cells and LPS‐treated macrophages.^113^, ^114^ Similarly, MSC‐derived extracellular vesicles suppress mitochondrial O_2_^.−^ levels in H_2_O_2_‐treated human fetal hepatocytes, which is associated with a reduction in apoptosis.^80^

The potential for MSCs to directly attenuate mitochondrial dysfunction has been demonstrated in an in vitro model of I/R injury in mouse ventricular myocytes.^54^ Within 5 minutes of reperfusion, cells exhibited an exaggerated Δψm hyperpolarization, which was reduced by conditioning the reperfusion solution with MSCs. The exaggerated hyperpolarization was followed by a continuous depolarization in controls after 15 minutes which was also attenuated by the paracrine secretion of MSCs. Decay of the Δψm was likely a result of the mitochondrial permeability transition pore opening. The exaggerated hyperpolarization of the Δψm was also averted by a mitochondrial ROS scavenger which simultaneously decreased mitochondrial O_2_^−^ generation demonstrating the close relationship between these events. Similarly, MSC secretion decreased mitochondrial O_2_^−^, which led to the suggestion that MSCs may also attenuate Δψm dysfunction via scavenging of O_2_^−^.

Depolarization of the Δψm in cisplatin‐treated renal proximal tubular cells has also been reportedly attenuated using exosomes derived from UC‐MSCs.^27^ In vitro, BM‐MSCs were demonstrated to upregulate uncoupling protein 2 (UCP2) transcription in H_2_O_2_‐treated alveolar basal epithelial adenocarcinoma cells which reduces the formation of mitochondria‐derived O_2_^−^ by lowering the proton‐motive force across the mitochondrial membrane and provides another potential mechanism for the alleviation of mitochondrial dysfunction.^100^ This was regulated by the paracrine secretion of stanniocalcin‐1 by MSCs, which enhanced UCP2, correlating with cell survival and decreased ROS generation. MSCs secreted stanniocalcin‐1 may also attenuate inflammation as it decreases mitochondrial ROS and subsequent activation of the nucleotide‐binding oligomerization domain (NOD)‐like receptor protein 3 (NLRP3) inflammasome.^111^ BM‐MSCs inhibited the activity of the NLRP3 inflammasome in primed macrophages which is responsible for recognizing damage‐associated molecular patterns and initiating the inflammatory cascade through activation and secretion of IL‐1β.^115^, ^116^ MSCs have also been demonstrated to secrete the redox‐sensitive protein DJ‐1, which has established roles in maintaining mitochondrial biogenesis and respiratory chain efficiency and could potentially mediate the neuroprotective effects of the MSC secretome as shown in Parkinson's disease models.^117^ Collectively, these studies demonstrate that MSCs can ameliorate mitochondrial dysfunction in a paracrine manner with diverse therapeutic outcomes.

### 4.4 Mitochondrial donation

Recently, a concept has emerged that MSCs may be able to alter oxidative phosphorylation and ROS generation in cells through donation of mitochondria themselves. Islam et al^118^ observed mitochondrial transfer from human BM‐MSCs to alveolar epithelium in a mouse model of LPS‐induced lung injury. BM‐MSC administration attenuated decreased intracellular ATP in the alveoli caused by lung injury; notably, ATP (visualized by a molecular probe) was predominantly restored at the site of mitochondrial transfer and immediately surrounding alveoli. MSCs with a mutation in connexin 43, a protein involved in the formation of gap junctions, were unable to transfer mitochondria despite being functionally competent and subsequently did not restore ATP, surfactant secretion, or reduce leukocyte infiltration. This phenomenon only occurred in LPS‐exposed lungs indicating that mitochondrial transfer is dependent on stimulants from damaged tissues.

MSCs cultured in hyperoxic (21% O_2_: normoxic atmosphere) conditions produce high levels of mitochondrial O_2_^.−^, depolarize Δψm, and induce mitophagy.^119^ Mitochondria are loaded into phagosomes and shuttled to the plasma membrane.^119^ These effects were reduced by culturing MSCs closer to a normoxic oxygen concentration (5% O_2_: hypoxic atmosphere). Macrophages have been observed to phagocytose these vesicles containing the partially depolarized mitochondria, which can fuse with endogenous mitochondria in macrophages. This protects silica‐exposed macrophages by increasing their oxygen consumption rate and decreasing mitochondrial O_2_^.−^ production. These effects could not be elicited when MSCs were substituted by human fibroblasts.^119^ This suggests that mitochondrial transfer may be stimulated by oxidative stress in MSCs. Conversely, MSCs have also been demonstrated to engulf mitochondria from other somatic cells exposed to H_2_O_2_. MSCs degraded the engulfed mitochondria which stimulated HO‐1 expression, mitochondrial biogenesis in MSCs, and the transfer of functional MSCs to damaged cells.^53^ Inhibition of mitophagy negated the cytoprotective effects of MSCs in other somatic cells, which suggests that MSC sensing of damaged mitochondria may mediate their therapeutic responses.^53^ Supporting this, the cell contact‐dependent transfer of mitochondria from chemotherapy‐treated T lymphocytes to MSCs was also determined to be critical to their ability to decrease mitochondrial O_2_^.−^ production and cell death in T lymphocytes.^120^ Nonetheless, in this study, mitochondrial transfer appeared to be predominately unidirectional and very few MSC‐derived mitochondria were observed in T cells. This suggests that MSC sensing of mitochondria can promote therapeutic mechanisms other than mitochondrial donation.^120^

Although extracellular vesicles can contain whole mitochondria, several studies suggest that the donation of mitochondria may be contact‐dependent. MSCs have been found to transfer mitochondria via tunneling nanotubes (TNT) to glucose‐deprived and hypoxia‐reoxygenated cardiomyocytes which prevented Δψm depolarization and cell apoptosis. Albeit inhibition of TNT formation only partially reversed the effects of MSCs indicating other cytoprotective mechanisms were still active.^121^ Similarly, MSCs transfer mitochondria to chemotherapy‐treated endothelial cells, which appears to occur in a unidirectional manner, unlike in T lymphocytes.^120^, ^122^ Miro1 is important to TNT formation and its overexpression in MSCs can enhance mitochondrial transfer.^123^ The effects of MSC mitochondrial donation are sufficient to rescue cellular respiration, proliferation, and motility in mitochondria‐depleted osteosarcoma cells.^124^ These effects can be maintained for 45 passages, which highlights the therapeutic potential of MSC‐derived mitochondria.^124^ Exogenous application of mitochondria isolated from MSCs may also offer therapeutic benefit and are able to protect dexamethasone‐treated muscle cells form oxidative stress in vitro.^125^ Albeit, delivery of MSC‐derived mitochondria in in vivo poses a challenge. While several studies have demonstrated contact‐dependent transfer of mitochondria between MSCs and other cells, MSCs have also been reported to donate mitochondria to LPS‐treated macrophages via secreted extracellular vesicles.^114^ Exposure of macrophages to MSC‐derived exosomes promoted their indication to the type 2 phenotype, which exerted anti‐inflammatory effects after adoptive transfer in septic lung injury.^114^ The effects of MSCs were dependent on enhancing mitochondrial function in macrophages and were inhibited by damaging mitochondria in MSCs and blocking extracellular vesicles.^114^ The therapeutic use of MSCs to deliver functional mitochondria to damaged tissue is an intriguing concept and warrants further study; however, another recent advancement reported by Panfoli et al^126^ suggests that the exosomes of MSCs are capable of oxidative phosphorylation independent of the mitochondria. Subsets of MSC‐derived exosomes isolated from the umbilical cord of term newborns were discovered to contain complexes of the ETC embedded in the membrane. These exosomes possessed an electrochemical membrane potential, consumed O_2_, and produced ATP. The therapeutic application of these exosomes is yet to be investigated; nonetheless, this may present a viable tool to restore dysfunctional oxidative phosphorylation and ATP synthesis in damaged cells.

## 5 CONCLUSION

The presented studies evidently demonstrate that MSCs exhibit antioxidant potential either directly via scavenging of ROS and donating mitochondria or indirectly by upregulation antioxidant defenses in other cells and altering cellular bioenergetics. These effects can occur in combination with the previously recognized trophic and vesicular components of the MSC secretome acting directly on regenerative pathways. Likewise, antioxidant and trophic pathways appear to mediate the cytoprotective effect of MSC treatments which are ROS dependent. MSCs have frequently been utilized in inflammatory diseases to modulate the immune response. In this context, immunosuppression can avert ROS generation which is generated by MPO and NOX enzymes as a part of the inflammatory response. However, MSCs have now been shown to exert immunosuppressive effects by dampening ROS production and enhancing mitochondrial function in macrophages and neutrophils. Therefore, the antioxidant properties confer a role in the trophic and anti‐inflammatory mechanisms of MSC therapy. Considering that oxidative stress is implicated in almost every disease, these antioxidant properties, along with regenerative capacity of MSC seretome, may explain why MSC treatments are useful for such a spectrum of seemingly unlinked pathologies (Figure 1). Future studies should seek to clarify disease‐specific nuances of the antioxidative mechanisms of MSCs and MSC‐derived products. Likewise, improving the antioxidant effects of MSCs by enhancing the expression of antioxidant enzymes or promoting mitochondrial donation may be useful to optimize MSC‐based therapies and improve outcomes.

## CONFLICT OF INTEREST

The authors declared no potential conflicts of interest.

## AUTHOR CONTRIBUTIONS

R.S.: conception and design, manuscript writing, final approval of manuscript. K.N.: conception and design, manuscript writing, financial support, final approval of manuscript.

## Data Availability

Data sharing is not applicable to this article as no new data were created or analyzed in this study.
